# The Relationship of Thyroid Cancer with Radiation Exposure from Nuclear Weapon Testing in the Marshall Islands

**DOI:** 10.2188/jea.13.99

**Published:** 2007-11-30

**Authors:** Tatsuya Takahashi, Minouk J. Schoemaker, Klaus R. Trott, Steven L. Simon, Keisei Fujimori, Noriaki Nakashima, Akira Fukao, Hiroshi Saito

**Affiliations:** 1 Department of Public Health, Yamagata University School of Medicine.; 2 Section of Epidemiology, Institute of Cancer Research.; 3 Gray Cancer Institute.; 4 Radiation Epidemiology Branch, National Cancer Institute.; 5 Division of Surgical Oncology, Graduate School of Medicine, Tohoku University.; 6 Department of Social Medicine, Nagasaki University School of Medicine.

**Keywords:** thyroid cancer, Marshall Islands, radioactive fallout, radiation exposure, Bikini Atoll

## Abstract

The US nuclear weapons testing program in the Pacific conducted between 1946 and 1958 resulted in radiation exposure in the Marshall Islands. The potentially widespread radiation exposure from radio-iodines of fallout has raised concerns about the risk of thyroid cancer in the Marshallese population. The most serious exposures and its health hazards resulted from the hydrogen-thermonuclear bomb test, the Castle BRAVO, on March 1, 1954. Between 1993 and 1997, we screened 3,709 Marshallese for thyroid disease who were born before the BRAVO test. It was 60% of the entire population at risk and who were still alive at the time of our examinations. We diagnosed 30 thyroid cancers and found 27 other study participants who had been operated for thyroid cancer before our screening in this group. Fifty-seven Marshallese born before 1954 (1.5%) had thyroid cancer or had been operated for thyroid cancer. Nearly all (92%) of these cancers were papillary carcinoma. We derived estimates of individual thyroid dose proxy from the BRAVO test in 1954 on the basis of published age-specific doses estimated on Utirik atoll and ^137^Cs deposition levels on the atolls where the participants came from. There was suggestive evidence that the prevalence of thyroid cancer increased with category of estimated dose to the thyroid.

The United States nuclear weapons testing program in the mid-Pacific was conducted between 1946 and 1958 on Bikini and Enewetak atolls in the Marshall Islands. A total of 66 nuclear tests resulted in radioactive fallout contamination of a number of neighborhood atolls. The potentially widespread exposure to radioactive iodines from nuclear fallout raised concerns about the excess risks of thyroid cancer in the Marshallese population (Figure).

**Figure.**
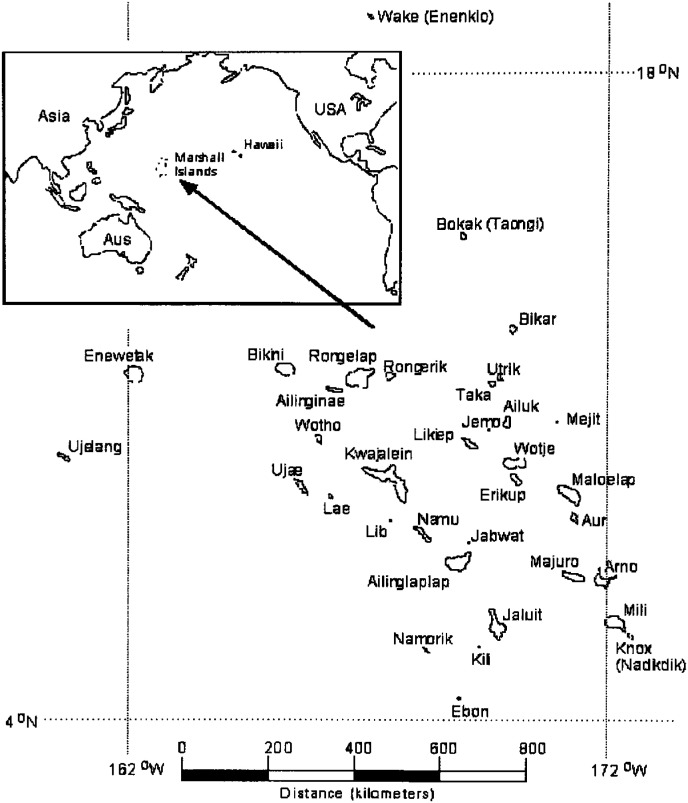
Map of the Marshall Islands

The radioactive fallout from the detonation of a hydrogen bomb on Bikini atoll on March 1, 1954, called the *Castle BRAVO*, cause the most serious radiation exposures to Marshallese people. An estimated average thyroid dose from the Bravo test in a one-year-old child was 52 Gy (5,200cGy) on the downwind atoll Rongelap and 6.8 Gy (680 cGy) on Utirik an atoll further downwind.^1^ These exposed communities of these two atolls were evacuated about 72 hours after the detonation and then provided with follow-up medical care over the decades since by the United States.^2^ The most frequent long-term health effect in the exposed population appeared to be an increased frequency of nodular thyroid disease including thyroid cancer.^3^ Twenty-three Japanese fishermen of Fukuryu Maru (Lucky Dragon) were also exposed to radioactive fallout from the BRAVO test nearby these two atolls. However, possibly exposed neighborhood atolls other than these two had been ignored.

A recent assessment by the Marshall Islands Nationwide Radiological Study has provided evidence that at least ten of the inhabited atolls or reef islands have been contaminated from the atmospheric explosions to various degrees.^4^^-^^6^ In conjunction with this assessment, we examined a large proportion of the Marshallese population potentially exposed to radioactive fallout for thyroid disease.^7^ We previously showed that thyroid nodules were very common, in particular in female Marshallese, and that the prevalence increased with age, but we found little evidence that benign thyroid nodules were related to exposure from nuclear fallout.^8^^-^^11^ In 2001, we published a monograph that summarized our previous findings systematically.^11^ In the monograph, we reported that the occurrence of thyroid cancer in Marshallese was still high in 40 years after the nuclear test and that the association between thyroid cancer prevalence and radio-iodines exposure using the data collected until the end of 2000.

Since the prevalence of radiation induced thyroid cancer was much different by sex and thyroid dose, it would be very interesting whether there was any difference, in the relationship between thyroid cancer prevalence and radiation dose, associated with sex and thyroid dose range.^12^ We had not analyzed the data restricted any particular subgroup in the monograph at all. We renew the entire data and analyze again using an alternate dose estimation that was more intuitive than that of the monograph. However, the shape of dose-response curve was still unclear, so that we would never discuss the relationship between thyroid cancer prevalence and restricted thyroid dose range in this paper.

Accordingly, we report new results in this paper: we investigate into the relationship between thyroid cancer prevalence and thyroid dose in particular subgroups defined by sex and conduct all analyses in this paper using alternate and more intuitive dose reconstruction than in the monograph.

## METHODS

One objective of this study was to determine the prevalence of thyroid cancer in Marshallese people who lived anywhere in the Marshall Islands during the atomic bomb testing period, in particular in 1954, and thus were potentially exposed to fallout from the nuclear weapons tests. Between 1993 and 1997, we examined 7,172 Marshallese people for thyroid disease during four study clinical phases of work. Of this group, 5,821 were born before January 1, 1965, and were included in the epidemiologic analysis reported here. Although the main population of interest was those alive during the testing period, we did not refuse anyone who requested an examination even if they were younger. Recruitment of the study participants was by public announcement, through radio, and by word of mouth. Attendance was very high due to much publicity in recent years about adverse health effects of radiation exposure. Among Marshallese, it resulted in great concern about long-term effects of the bomb testing and about thyroid disease in particular. In the clinical phases, we examined 1,603 people in Ebeye, Kwajalein Atoll (1993 and 1996), 5,255 people in Majuro and Utirik Atolls (1994) and 314 people on two outer (remote) atolls and a reef island, namely Likiep, Ailuk and Mejit (1997). Many residents of Ebeye and Majuro, two of the biggest population centers in the Marshall, had previously lived on other outer atolls.

The thyroid screening program was composed of two components: a personal interview and a clinical examination. Every participant who gave informed consent, after having been explained the background and the purpose of the study, was interviewed by a Marshallese assistant in his or her native language and underwent a medical examination by a medical team of us. The interview consisted of questions relating to date of birth, residence history, reproductive history, general health and diet. The questionnaire inquired about atoll of residence for each year from birth to the year of interview. This information was cross-checked by asking for date and place of marriage and date and place of births of children of the study participants.

The clinical examination consisted of a medical examination of the thyroid by a physician including palpation of the neck and an ultrasound examination of the thyroid by a second physician using an ALOKA echo camera SSD-121™ (ALOKA Co. Ltd., Tokyo) with a 7.5 MHz mechanical sector probe. Both physicians were blinded to the results of the interview. For purposes of the examinations and our analysis, we defined a nodule as a focal abnormality of the echo pattern that was larger than 4 mm. All participants identified with a palpable nodule were further examined by a fine needle aspiration biopsy of the dominant nodule. All medical examination methods were reviewed and approved in 1992 by the Institutional Review Board of the Marshall Islands Health Services.

Medical findings from thyroid examinations were reported to individuals and local health services. If a scar in the neck was found during the medical examination that suggested past history of any thyroid surgery, we asked details of the surgery and then attempted to retrieve the histopathological report. If thyroid cancer was suspected on the basis of the ultrasound examination and the fine needle aspiration biopsy results, thyroid surgery was conducted in Majuro Hospital by our team during a special visit for surgery. Histopathological specimens from surgery were assessed in the Department of Pathology of Tohoku University Hospital, Sendai. The medical monitoring has been described in detail elsewhere.^8^^,^^11^

Three study populations (birth cohorts) were defined: those born before the BRAVO test on March 1, 1954 (*Bravo cohort*), those born between March 1, 1954 and the end of the bomb test period (*end-of-testing cohort*), and those born between March 1, 1959 and January 1, 1965 (after testing cohort), the latter group to serve as unexposed control. The end-of-testing cohort included people who were *in utero* for more than three months at the end of the last atomic bomb detonation in August 1958 and thus were born before March 1, 1959 since the fetal thyroid actively concentrates iodine from the fourth month of gestation.^13^

Since at present no detailed assessment of individual radiation dose to the thyroid gland has been conducted for people in the study, and there is very little other information about received doses in the Marshall Islands, we based our analysis on surrogate measures of radiation dose. The only data on radiation dose from fallout in the Marshall Islands come from Lessard et al.,^1^ who estimated thyroid doses for people who were exposed to radioactive fallout on Rongelap and Utirik after the BRAVO test. For our surrogate measures of dose, we utilized these estimates by Lessard et al. and information on ^137^Cs soil deposition levels from the Republic of the Marshall Islands Nationwide Radiological Study carried out by one of the authors.^4^^-^^6^

Dose estimates for newborns and for persons 1, 6, 9, 12, 14, and 20 years old on Utirik have been published. The dose on Utirik was estimated to be 59 cGy to newborns, 680 cGy to one-year-olds, 350 cGy in six-year-olds, and was an average of 165 cGy for people over age 20.^1^ We derived dose estimates for other ages from age one onwards by fitting a fractional polynomial model^14^ that allowed us to interpolate between ages.

Unfortunately, we found it necessary to omit people exposed in the first year of life because we felt that it was not possible to attribute a credible thyroid dose to this age group for lack of any information on possible ingestion pathways and because the estimate for new-borns by Lessard et al. did not fit with the age-dose relationship for the older age groups. Maximum ^137^Cs soil deposition levels for each atoll, decay-corrected to time of deposition, were taken to be representative of the original deposition. Hence, we used those values to scale (weight) the age-specific dose from Utirik to other atolls.^6^ The highest maximum ^137^Cs soil deposition was observed on Bikini Atoll (906,000 Bq/m^2^). The deposition levels were 11,100 on Utirik, 5,210 on Ailuk, 4,820 on Mejit, 3,660 on Likiep, 518 on Kwajalein and 198 on Majuro. Thus, the thyroid dose on Ailuk was estimated about a half of that on Utirik. The deposition level was nearly constant among the atolls within the latitude range of 4°N to 9°N and was shown to increase sharply with increasing latitude between 9° and 11.5°N where Bikini and Enewetak Atolls are located (Figure).

People who reported to be outside the Marshall Islands in 1954 were assumed unexposed. Sixteen people reported to have lived on more than one atoll in the year 1954. For two of them, because the ^137^Cs levels were similar between the atolls, we could obtain an estimate of weighted Utirik dose. We excluded people who reported to have lived on Bikini or Enewetak in 1954 since this obviously implied an error in the residence history; nobody was allowed to live on these atolls during the testing. We also had to exclude people on Rongelap, because the absorbed doses were very high and probably exceeded the linear part of the dose response curve. In addition, the employed method assumed that the ^137^Cs deposition levels were proportional to iodine transfer; this assumption was unlikely to be met on Rongelap because the atoll is very close to the test site.

We conveniently call this crude estimate “Weighted Utirik dose.” We use this proxy of thyroid dose only for ordering study participants according to their thyroid dose since there would be still substantial uncertainty in this measure. Subsequently, we do not discuss the shape of dose-response curve *per se* since the dose-response curve derived from weighted Utirik dose might be misleading.

The prevalence of thyroid cancer was described by histological type, age at diagnosis and according to study birth cohort, sex, and period of birth within the cohorts. Logistic regression analysis was used to model the odds of having thyroid cancer according to the weighted Utirik dose (quartiles based on non-cancer subjects), age at exposure (5 year age-bands), and sex. In addition, the analysis was conducted by sex. Analyses were carried out using the statistical program Stata.^15^ All statistical tests were two-sided and significance was defined as p<0.05.

## RESULTS

Our findings pertain specifically to 5,821 Marshallese that we examined between 1993 and 1997 and who were born before January 1, 1965. Among this group were 4,762 Marshallese born before the end of the testing period and thus potentially exposed, with 3,709 born before the BRAVO detonation. In 1999 census of the Marshall Islands, the population of 45 years of age or older in the Marshall Islands was 5,970 (i.e. 5,970 peoples were born before 1954 and were alive in 1999). In 1993-1997, target population was estimated 6,000 or more, so that our examined group corresponds to about 60% of the entire population at risk and who were still alive at the time of these examinations.^16^

In our study sample, we found 659 who had palpable thyroid nodule. Six hundreds and thirty-eight out of 659 (97%) underwent fine needle aspiration biopsy. From these subjects, we newly diagnosed 38 thyroid cancers, 24 of which were papillary carcinomas, 8 micropapillary carcinomas and 6 follicular carcinomas (Table 1). All of these cases were operated by our team in Majuro in 1994 and in 1996. No severe complication of surgery occurred. Operated patients were followed-up every two or three years after surgeries. All of them were alive without any evidence of recurrence of thyroid cancer until 2002.

**Table 1.** Histology and age distribution of thyroid cancer.

Characteristic	Diagnoses from this work		Diagnosed prior		Total	


*Thyroid cancer histology^a^*						
Papillary	24	(63)	16	(53)	40	(59)
Micropapillary	8	(21)	3	(10)	11	(16)
Follicular	6	(16)	1	(3)	7	(10)
Follicular + papillary	0		1	(3)	1	(2)
Unspecified	0		9	(30)	9	(13)

*Age at diagnosis (years)^b^*						
<20	0		2	(7)	2	(3)
20-30	1	(3)	3	(10)	4	(6)
30-39	9	(24)	11	(37)	20	(29)
40-49	11	(29)	10	(33)	21	(31)
50-59	8	(21)	2	(7)	10	(15)
60-69	6	(16)	1	(3)	7	(10)
70+	3	(8)	0		3	(4)
Not Known	0		1	(3)	1	(2)

Total	38		30		68	

^a^ p = 0.26 for the test of homogeneity excluded unspecified cancer

^b^ calculated on the basis of year of surgery for people diagnosed prior to the study, p = 0.05 for the test of homogeneity excluded age not known

Percentages in parentheses.

In addition, we found 106 study participants who had scars that suggested previous surgery in the neck region. Among those were 16 papillary carcinomas, 3 micropapillary carcinomas, 1 follicular carcinoma, 1 mixed follicular/papillary carcinoma and 9 unspecified thyroid cancers. Our analysis of prevalence of thyroid cancer is based on a total of 68 cases of thyroid cancer. Histological reports of non-cancer cases frequently cited as adenomatous goiter (32 cases), follicular adenoma (8 cases) and other benign nodule (31 cases). Seventy five percent of the thyroid cancers were diagnosed before age 53 years. For 5 previously operated people, the histopathological report could not be retrieved.

The prevalence of ever having had thyroid cancer was 1.2% (68/5,821) in our entire study group. Thyroid cancer was more common among females, 1.5% (46/3,147) versus 0.8% (22/2674) in males (Table 2). The prevalence was highest in the Bravo cohort (1.5% [57/3709]) with a prevalence of 2.1% (40/1922) in females and 1.0% (17/1787) in males. There was no indication in this cohort that the prevalence was higher in the people who were exposed as children compared with those who were exposed as adults. Thyroid cancer was less common in the end-of-testing and after testing cohorts. However, since in our study all subjects were examined in 1973-1997, the younger at exposure was the younger in the examination period. This fact resulted in the difficulty of partitioning age at exposure effect from attained age effect in our study.

**Table 2.** Prevalence of thyroid cancer by calendar year of birth.

Period of birth	Male		Female		Total	

	This work	Prior diagnosed	This work	Prior diagnosed	This work	Prior diagnosed
*Bravo cohort*						
Before 1936	4	1/556	6	7/561	10	8/1117
(Adults in 1954)	(0.72)	(0.18)	(1.07)	(1.25)	(0.90)	(0.72)
1936-1945	4	4/397	3	4/467	7	8/864
(Teenagers in 1954)	(1.01)	(1.01)	(0.64)	(0.86)	(0.81)	(0.93)
1945-February 1954	3	1/834	10	10/894	13	11/1728
(Children in 1954)	(0.36)	(0.12)	(1.12)	(1.12)	(0.75)	(0.64)

Total for Bravo cohort	11	6/1787	19	21/1922	30	27/3709
	(0.62)	(0.34)	(0.99)	(1.09)	(0.81)	(0.73)

*End-of-testing cohort*	2	2/444	10	0/609	4	2/1053
(March 1954 -February 1959)	(0.45)	(0.45)	(1.64)	(0)	(0.38)	(0.19)

*After testing cohort*	1	0/443	5	1/616	4	1/1059
(March 1959-January 1965)	(0.23)	(0)	(0.81)	(0.16)	(0.38)	(0.09)

Total for the three cohorts	14	8/2674	24	22/3147	38	30/5821
	(0.52)	(0.30)	(2.92)	(0.70)	(0.65)	(0.52)

Percentages in parentheses.

Weighted Utirik doses were derived for 3,378 people (91%) of the Bravo cohort. Fifty-six people were omitted because they reported to have lived on the highly exposed atolls of Bikini, Enewetak or Rongelap in 1954, 83 people because they reported to have lived on multiple atolls or because their atoll of residence was not known, and 194 people because they were in the first year of life on March 1, 1954. In the last case, dose estimates of this age group were highly uncertain due to lack of information on diet for quantifying exposure pathways. For two of subjects lived on multiple atolls, because the ^137^Cs levels were similar between the atolls, we could obtain an estimate of weighted Utirik dose. Among those omitted people, seven cases of thyroid cancer were known (three were on Rongelap at the time of the BRAVO test, atoll of residence in 1954 was unknown for two, and two was under age one year).

The weighted Utirik dose varied from 676 cGy in one-year-olds on Utirik at the time of BRAVO, to virtually zero for all ages in Namorik, Lib and Jabwat where the ^137^Cs deposition level did not exceed that from global fallout. Women in the third and fourth quartiles of weighted Utirik dose had the highest prevalence, 2.4% and 2.5% respectively (Table 3). The prevalence in the third and fourth quartiles remained high even when we excluded the women who were exposed on Utirik (2.4% and 1.8%, respectively). The prevalence of thyroid cancer appeared to increase with quartile of weighted Utirik dose in female (Table 4), but not in males (Table 5). In the regression model adjusted for age at the BRAVO test and sex, subjects in the fourth quartile of weighted Utirik dose had a non-significant 1.7-fold (1.67; 95% Confidence Interval [CI] 0.73-3.83) increased risk of having had thyroid cancer compared to subjects in the lowest quartile of dose (Table 3). The odds ratio of thyroid cancer increased with thyroid radiation dose, but the trend was not statistically significant (p = 0.15, two-sided). In addition, females had two-fold higher risk of thyroid cancer than males (2.11; 95% CI 1.14-3.89).

**Table 3.** Risk factors of thyroid cancer among 3,378 people alive at the Bravo test for whom a weighted Utirik dose could be derived.

	No. of cases of thyroid cancer (%)			No. of people			Adjusted odds ratio^a^ for total (95% CI)

Factor	Male	Female	Total	Male	Female	Total	
*Weighted Utirik dose (median) cGy*							
0 - 3.41 (2.33)	3 (0.7)	8 (1.9)	11 (1.3)	414	429	843	1.00
3.42-7.47 (5.56)	6 (1.3)	4 (1.1)	10 (1.2)	460	382	842	0.99 (0.41-2.42)
7.48 - 18.71 (10.23)	2 (0.6)	12 (2.4)	14 (1.7)	345	501	846	1.37 (0.59-3.14)
18.72 - 676.66 (77.00)	4 (1.0)	11 (2.5)	15 (1.8)	413	434	847	1.67 (0.73-3.83)
							*χ*^2^ trend=2.03
							p=0.15

*Age at the time of Bravo (median) years*							
1-4.9 (3.14)	1 (0.2)	6 (1.4)	7 (0.8)	417	416	833	1.00
5-9.9 (7.25)	2 (0.6)	12 (2.9)	14(1.8)	348	416	764	2.38 (0.94-5.99)
10-14.9 (12.11)	4 (1.7)	3 (1.2)	7 (1.4)	239	249	488	1.94 (0.66-5.64)
15-19.9 (17.65)	5 (2.9)	4 (1.9)	9 (2.4)	170	206	376	3.26 (1.18-9.03)
20+ (26.84)	3 (0.7)	10 (2.2)	13 (1.4)	458	459	917	2.04 (0.78-5.34)
							*χ*^2^ trend=1.70
							p=0.19

All dose categories	15 (0.9)	35 (2.0)	50 (1.5)	1,632	1,746	3,378	Odds ratio for sex (Female/Male)
							2.11 (1.14-3.89)

^a^ This model included weighted Utirik dose, age at the BRAVO and sex

CI: confidence interval.

**Table 4.** Risk factors of thyroid cancer among 1,746 females alive at the Bravo test for whom a weighted Utirik dose could be derived.

Factor	No. of cases of thyroid cancer (%)	No. of people	Adjusted odds ratio^a^ (95% CI)


*Weighted Utirik dose (median) cGy*			
0 - 3.41 (2.33)	8 (1.86 %)	429	1.00
3.42-7.47 (5.29)	4 (1.05 %)	328	0.54 (0.15-1.92)
7.48 - 18.71 (9.50)	12 (2.40 %)	501	1.36 (0.53-3.49)
18.72 - 676.66 (73.82)	11 (2.53 %)	434	1.50 (0.56-3.99)
			*χ*^2^ trend=1.83
			p=0.18

*Age at the time of Bravo*			
years			
1-4.9	6 (1.44 %)	416	1.00
5-9.9	12 (2.88 %)	416	2.43 (0.89-6.63)
10-14.9	3 (1.20 %)	249	1.02 (0.25-4.16)
15-19.9	4 (1.94 %)	206	1.57 (0.43-5.78)
20+	10 (2.18 %)	459	1.71 (0.58-4.98)
			*χ*^2^ trend=0.17
			p=0.68
*All categories*	35 (2.00 %)	1,746	

^a^ This model included weighted Utirik dose and age at the BRAVO

CI: confidence interval.

**Table 5.** Risk factors of thyroid cancer among 1,632 males alive at the Bravo test for whom a weighted Utirik dose could be derived.

Factor	No. of cases of thyroid cancer (%)	No. of people	Adjusted odds ratio^a^ (95% CI)


*Weighted Utirik dose (median) cGy*			
0 - 3.41 (2.40)	3 (0.72 %)	414	1.00
3.42-7.47 (5.74)	6 (1.30 %)	460	2.11 (0.51-8.80)
7.48 - 18.71 (11.60)	2 (0.58 %)	345	0.96 (0.15-6.05)
18.72 - 676.66 (83.98)	4 (0.97 %)	413	2.02 (0.42-9.66)
			*χ*^2^ trend=0.28
			p=0.59

*Age at the time of Bravo years*			
1-4.9	1 (0.24 %)	417	1.00
5-9.9	2 (0.57 %)	348	2.43 (0.21-27.52)
10-14.9	4 (1.67 %)	239	7.14 (0.77-66.40)
15-19.9	5 (2.94 %)	170	14.62 (1.63-131.52)
20+	3 (0.66 %)	458	2.96 (0.29-30.75)
			*χ*^2^ trend=1.86
			p=0.17

*All categories*	15 (0.92%)	1,632	

^a^ This model included weighted Utirik dose and age at the BRAVO

CI: confidence interval.

In females, subjects in the fourth quartile had a non-significant 1.5-fold (1.50; 95% CI 0.56-3.99) risk of having had thyroid cancer compared to the lowest quartile but the trend also was not statistically significant (p = 0.18) (Table 4). On the other hand, in males there was not any substantial trend was observed while subjects in the fourth quartile of weighted Utirik dose had a 2.0-fold (2.02; 95% CI 0.42-9.66) increased risk of having had thyroid cancer compared to subjects in the lowest quartile of dose (Table 5).

## DISCUSSION

The prevalence of thyroid cancer is high in the Marshall Islands. Among people, we examined and who were alive at the time of the BRAVO test, about one male and two females per 100 residents were diagnosed with thyroid cancer or had been operated for thyroid cancer prior to our study. The prevalence is increased in subjects who were potentially exposed to radio-iodines from the atomic weapon testing program, but is still 0.5% in people who were born afterwards. Previous research on radiation-induced thyroid cancer has documented an increasing risk of thyroid cancer with younger age at exposure.^17^^-^^19^ We did not find any evidence of any pronounced influence of age at exposure on thyroid cancer prevalence in our data. However, in our study all subjects were examined within relatively short period (1973-1997), so that the younger at exposure was the younger at the examination. In this study, it would not be possible to partition age at exposure effect from attained age effect.

The age distribution of papillary cancer in an unexposed the U.S. population showed that 60% of the patients were between age 30 to 59 years, with a peak at 43 years-old.^20^ Papillary cancer was more common among young people than among elderly, 15% of the patients were between 20 and 29 whereas only 5% of the patients were older than 70 years. That age distribution is very similar to that among the Marshallese described in Table 1. Our interpretation is that the thyroid cancers in the Marshallese population are likely to be a mixture of those that are radiation-induced and those that have occurred for other, unknown reasons (naturally occurring cancers). Moreover, since most thyroid cancers were diagnosed from a rather brief period of active screening, there is a strong link between age at exposure and age at diagnosis.

The prevalence of thyroid cancer in the Bravo cohort increased with quartile of weighted Utirik dose in all subjects and in females, though the test for trend was not significant. The use of the crude dose estimates could have easily diluted any real trend of thyroid cancer prevalence with increasing thyroid dose in part, due to exposure misclassification. It was likely that misclassification could affect in lower dose range since the dose of first two quarterlies were not much different from the global (background) radiation dose. There is significant scope for improvement of this analysis by using dosimetric estimates that is more precise. The dose response relationship that we observed looks similar to that found during the follow-up investigations of the populations of Rongelap and Utirik.^21^ The incidence of papillary thyroid cancer was highest in the Rongelap and Ailingnae population (5/86; 5.8%). In the less exposed Utirik population, it was 4/167 (2.4%), and in the presumably unexposed control population, it was 2/227 (0.9%). However, in our study, this dose response relationship was also seen among Marshallese people who were not on Rongelap or Utirik in 1954 at the time of the BRAVO test.

Susceptibility to thyroid cancer might be modified by genetic background. About 3-6% of the thyroid cancer cases in Europe and the United States show familial aggregation.^22^ Populations at higher risk include Moroccans and people with Jewish ancestry.^12^ The highest thyroid cancer incidence has been observed in Melanesians in New Caledonia.^23^ It still needs to be established whether Marshallese people are at increased risk because of genetic makeup. The other important modifier of thyroid cancer risk in the Marshall Islands is iodine deficiency. We reported that a moderate degree of iodine deficiency was observed which might be responsible for some of the increased prevalence of thyroid nodules.^9^^,^^11^ However, it is possible that iodine deficiency observed in our past study, only reflected present iodine intake status, was a recent event for Marshallese. In their traditional life-style that has drastically changed into the Western style for the last decade, Marshallese had eaten local reef fish and coconut daily and seemed to be guaranteed to sufficient iodine intake.

The limitations of this study were uncertainty of thyroid radiation dose reconstruction and possible self-selection of the study participants. We made two important but limiting assumptions with our method of thyroid dose estimation. First, we assumed that only the tests conducted in 1954, and in particular, the BRAVO test, contributed to thyroid dose. There were 66 detonations over 13 years but 1954 was the most important year of the nuclear weapon testing program since the BRAVO detonation together with five other tests produced 45% of the total yield (equal to 107 MT equivalent TNT) from all tests during the program.^4^ The BRAVO test was also the only test that necessitated immediate evacuation of the people living in surrounding atolls, namely Rongelap and Utirik. The second assumption was that observed ^137^Cs soil deposition levels, resulting from all tests, were proportional to iodine transfer from the tests conducted in 1954.

The estimated participation proportion was about 60%. Then, it was possible that the participation related to the exposed radiation dose. However, this self-selection bias might be small because the results from the analysis of benign thyroid nodules among the same subjects did not show any relationship between the prevalence and thyroid dose.^11^

The data presented here suggest a dose dependent increase of thyroid cancer prevalence among Marshallese who were living on other atolls than Rongelap and Utirik. To improve the certainty of this conclusion, individual thyroid dose reconstruction becomes an essential task for future work.
